# Preparation, evaluation and metabolites study in rats of novel amentoflavone-loaded TPGS/soluplus mixed nanomicelles

**DOI:** 10.1080/10717544.2019.1709920

**Published:** 2020-01-08

**Authors:** Xue Feng, Yuting Chen, Luya Li, Yuqian Zhang, Lantong Zhang, Zhiqing Zhang

**Affiliations:** a Department of Pharmaceutical Analysis, School of Pharmacy, Hebei Medical University, Shijiazhuang, PR China;; b The Second Hospital of Hebei Medical University, Shijiazhuang, PR China

**Keywords:** Amentoflavone, nanomicelle, cytotoxicity, cellular uptake, metabolite, UHPLC-Q/TOF-MS

## Abstract

Amentoflavone (AMF) is a kind of biflavonoids existing in Ginkgo biloba leaves. It has many biological activities, such as antioxidant, anti-inflammatory, anti-bacterial, antiviral, hypoglycemic, anti-tumor and inducing apoptosis. However, its solubility and bioavailability are poor and there are a few studies on it *in vivo*. In this study, to improve its solubility and bioavailability, the nanomicelles were prepared with TPGS and soluplus as carriers for the first time. The particle size, Zeta potential, encapsulation efficiency, drug loading, stability, cytotoxicity, cellular uptake, and metabolites in rats were studied. Cytotoxicity, cellular uptake, and metabolites in rats of AMF-loaded TPGS/soluplus mixed micelles were compared with those of AMF. As a result, AMF-loaded TPGS/soluplus mixed micelles with a particle size of 67.33 ± 2.01 nm and Zeta potential of −0.84133 ± 0.041405 mV were successfully prepared. The encapsulation efficiency and drug loading of the mixed nanomicelles were 99.18 ± 0.76% and 2.47 ± 0.01%, respectively. The physical and chemical properties of the mixed micelles were stable within 60 d, and the cytotoxicity of the mixed micelles was much greater than that of AMF monomers. Thirty-four kinds of metabolites of AMF were identified in rats. The metabolites were mainly distributed in rat feces. No metabolites were detected in bile and plasma. 14 kinds of metabolites of the mixed micelles in rats were detected, including 11 in feces, 6 in urine, and 3 in plasma, which indicated that the bioavailability of AMF has been improved. And the toxicity to cancer cells was enhanced, which laid a foundation for the development of new drugs.

## 1. Introduction

Ginkgo is a deciduous tree of ginkgo family and ginkgo genus. It is an ancient gymnosperm with a growth history of several hundred million years (Gong et al., 2008). Ginkgo biloba leaves are rich in more than 200 kinds of compounds such as lactones, polysaccharides, flavones, organic acids and phenolic acids (Ude et al., 2013). Biflavonoids as special flavonoids, their activities are higher than that of monoflavonoids in some aspects. Therefore, a more detailed study on biflavonoids has a good application prospect and significance. As a kind of biflavonoids in ginkgo biloba leaves, amentoflavone (AMF) has many biological activities, such as antioxidant (Zhang et al., 2015; Lee & An, 2016), anti-inflammatory (Zhang et al., 2015), antifungal (Hwang et al., 2012), antiviral (Coulerie et al., 2013), hypoglycemic (Su et al., 2019), anti-tumor (Guruvayoorappan & Kuttan, 2008), and inducing apoptosis (Pei et al., 2012; Zhaohui et al., 2018).

Studies have shown that the metabolism and elimination of AMF in rats are fast, and the bioavailability of AMF by intraperitoneal injection was 77.4%± 28%, but the oral bioavailability is very low (Liao et al., 2015; Yu et al., 2017). Researches on the tissue distribution of AMF in rats showed that after oral administration, the drugs are mainly distributed in the small intestine and stomach, followed by the liver and large intestine, and only a small part can enter other tissues with blood circulation. And it is difficult to dissolve in water and organic solvents, so it is difficult to be absorbed by the body. To give full play to the pharmacological effects of AMF, bioavailability and solubility must be improved. The main methods to improve the bioavailability and solubility of drugs are to change the way of drug delivery, change the dosage form (Wei et al., 2014; Hu et al., 2017; Santos et al., 2019) or modify its structure (Sen Gupta & Ghosh, 2017). Because it is difficult for AMF to dissolve in water and organic solvents, it is almost impossible to change the way drugs are administered. In recent years, micelle-based drug delivery systems have been widely developed because of their enhanced pharmacokinetics, biological distribution and high stability (Kesharwani et al., 2018; Zhang et al., 2019), and oral bioavailability has been greatly improved after the preparation of micelles (Guo et al., 2017). So in this experiment, the nanomicelle was made, and evaluated *in vitro* and *in vivo*.

As everyone knows, when a drug enters the body, a series of metabolites are produced, and then excreted in urine and feces. In the series of biotransformation processes, there are four aspects of pharmacological consequences: (1) transforming into inactive substances; (2) transforming the drug with no pharmacological activity into active metabolites; (3) changing the types of pharmacological actions of drugs; (4) producing toxic substances (Yuan et al., 2014; Liao et al., 2018). Therefore, it plays an important role to study the changes of drugs *in vivo* to make sure the safe and rational use of new drugs. In addition, the study of metabolites can also reflect the absorption of drugs in the body. In this experiment, the metabolites of the biflavone were analyzed in detail, which laid a foundation for the pharmacological research and the development of new drugs.

Mixed micelles are self-assembled micelles of two or more chemical materials and drugs with nano-size. Mixed micelles have many advantages over single micelles. First, they can encapsulate drugs in the core region to prevent degradation of drugs by external substances, such as gastric acid and cytochrome P450, resulting in the improvement of their stability (Nishiyama & Kataoka, 2006; Chiappetta & Sosnik, 2007). Second, these carriers are nanostructures formed by self-assembly of amphiphilic copolymers in aqueous media, exposing hydrophilic tails outside and hiding the hydrophobic head in the core region. Consequently, the solubility of hydrophobic drugs is increased (Moretton et al., 2014). Third, the mixed micelle system can reduce the inconsistency and nonspecific uptake of reticuloendothelial system, and enhance the targeting of drugs by enhancing permeability and retention effect (Gaucher et al., 2005). In addition, the mixed micelles range from 20 to 200 nm is large enough to avoid rapid elimination of renal tubules, and also small enough to penetrate blood vessels, enhance drug targeting and reduce toxicity to nonspecific organs. Besides, mixed micelles can also reduce adverse effects of drugs, increase drug loading and delay drug release, so they have been widely used in recent years, which has considerable research prospects (Bernabeu et al., 2016; Xu et al., 2016).

In recent years, researchers have studied a large number of mixed micelles by combining the outstanding advantages of different types of single micelles (Jain & Kumar, 2010; Jiang et al., 2019). In this study, polyvinyl caprolactam–polyvinyl acetate–polyethylene glycol graft copolymer (soluplus) and d-α-tocopheryl polyethylene glycol 1000 succinate (TPGS) were used as carriers to prepare mixed micelles of AMF. Soluplus has good solubilization for the drugs with poor water solubility, and can reduce the critical micellar concentration (CMC) value (0.76 × 10–3% w/v) and increase the drug loading (Bernabeu et al., 2016; Hou et al., 2019). However, in recent years, there are few studies on soluplus to improve the solubility of drugs, so the research prospect of soluplus is very broad. TPGS is widely used in the preparation of pharmaceutical formulation products as solubilizing agent, granulation aid, emulsifying agent, surfactant, ointment base, and suspending agent in pharmaceuticals (Guo et al., 2013; Koulouktsi et al., 2019). And there were also researches showed that it can inhibit the effect of P-gp, and promote cell apoptosis and show certain toxicity to cancer cells (Varma & Panchagnula, 2005; Neophytou et al., 2014; Bernabeu et al., 2016).

Nanoparticle drugs of oral administration have to overcome the mucosal diffusion barrier and epithelial absorption barrier except ensure their integrity in the gastrointestinal tract. This requires the particle surface relatively electro-neutral and have a particle size of less than 200 nm (Wu et al., 2019). Therefore, this experiment was dedicated to the preparation of mixed nanomicelles with electro-neutral surface, a particle size of less than 200 nm, high solubility, and good stability. Due to its low content in nature, the current research on AMF was mainly limited to its pharmacological activity, less research on its *in vivo* process, and no research on its preparation. Therefore, this experiment would fill the gaps in these aspects.

Recently, cancer is one of the most common lethal diseases in the world. The main treatment methods are surgery, chemotherapy and radiotherapy. But the treatment results are not completely satisfactory, the cure rate and the life quality of patients is low. In these days, researchers all over the word make every effort to develop new drugs that can treat cancer effectively. AMF is a biflavonoids composed of two molecules of apigenin by C–C bonds. Due to its low toxicity to normal cells and its anti-cancer effect, it is expected to have a better effect in the treatment of cancer. Because nanomedicines have good targeting and good solubility in the treatment of cancer, the preparation of nanomicelles with low toxicity to normal cells into nanomicelles can reduce side effects and enhance drug stability, prolong the release time and can also increases its targeting and toxicity to cancer cells (Wan et al., 2018), so it has a good development prospect.

## 2. Materials and methods

### 2.1. Instruments

Triple TOF™ 5600 + high resolution tandem mass spectrometry (AB SCIEX, Redwood City, CA); Ultra-high performance liquid chromatography system (Shimazhu 20A, Shimadzu Corporation, Tokyo, Japan); D3024R bench refrigerated centrifuge (Beijing Dalong Co., Ltd., Beijing, China); EYELLA N1100 rotary evaporator (Tokyo Rikakikai Co., Ltd., Tokyo, Japan); Ultrasonic crushing instrument (Wuxi Voshin instruments Manufacturing Co., Ltd., Huishan, China); Nano-ZS particle size tester (Malvern Instruments, Worcestershire, UK); Ultimate3000 high performance liquid chromatography (Thermo Fisher Scientific, Waltham, MA); pectraMax Plus384 Molecular Devices (Molecular Devices, Silicon Valley, CA); HF240 cell culture box (Shanghai Lishen Scientific Instrument Co., Ltd., Shanghai, China); SW-CJ-2FD clean bench (Suzhou Antai Airtech Co., Ltd, Beijing, China); T9S dual-beam ultraviolet-visible spectrophotometer (Beijing Persee General Instrument Co., Ltd, Beijing, China); FV1200MPE laser confocal microscope (Olympus, Tokyo, Japan).

### 2.2. Chemicals and materials

AMF (18040241, purity > 98%) was purchased from Shanghai Shifeng Biological Technology Co., Ltd., Shanghai, China. Soluplus was purchased from BASF (Ludwigshafen, Germany). Vitamin E polyethylene glycol succinate (TPGS) was purchased from Shanghai Yuanye Bio-Technology Co., Ltd., Shanghai, China. HPLC-grade methanol and acetonitrile were purchased from American *J.*T.-Baker Chemical Company (Phillipsburg, NJ). HPLC-grade formic acid was provided by Diamond Technology (Dikma Technologies Inc., Lake Forest, CA). CCK-8 was purchased from Beijing Zoman Biotechnology Co., Ltd., Beijing, China. Dimethyl sulphoxide (DMSO), iodine (I_2_), potassium iodide (KI) and sodium carboxymethyl cellulose (CMC-Na) were purchased from Tianjin Yongda Reagent Co., Ltd., Tianjin, China. Pure water was purchased from Hangzhou Wahaha Group Co., Ltd., Zhejiang, China.

### 2.3. Methods

#### 2.3.1. Preparation of mixed micelle

The preparation of AMF-loaded TPGS/soluplus mixed micelles was carried out by membrane hydration method (Zhao et al., 2017). Briefly, AMF (2 mg), soluplus (60 mg), and TPGS (20 mg) were dissolved in 20 mL methanol in a round bottom flask. Then, the solvent was evaporated by rotary evaporation to obtain a thin film. Subsequently, 8 mL water was added in the round bottom flask and hydration by ultrasonic for 1 h to obtain a clear micelle solution. After that, it was ultrasonic crushed for 5 min. Finally, the micelles were filtered with 0.22 μm filter membrane to remove unencapsulated drug. The blank micelles were prepared by the same method.

#### 2.3.2. Determination of critical micelle concentration (CMC)

The critical micelle concentration (CMC) is an important indicator for evaluating the stability of micelles. The lower the CMC, the more stable the nanomicelles. In this experiment, the CMC of AMF-loaded TPGS/soluplus mixed nanomicelles was measured using the iodine hydrophobic probe method and determined by an ultraviolet spectrophotometer. To prepare I_2_/KI standard solutions, 0.5 g of I_2_ and 1 g of KI were dissolved in 50 mL of deionized water. Then different ratios of TPGS/soluplus solutions with a concentration of 0.00001–0.2% were prepared, and 25 μL of I_2_/KI standard solution was added to each sample. Next, the mixtures were equilibrated at room temperature for 12 h in the dark. Finally, the ultraviolet absorbance value of each variant polymer concentrations were measured at 366 nm by a UV spectrophotometer. Plot the absorption intensity against the logarithm of the polymer mass concentration. When the absorbance increased sharply, the concentration of the micelle carrier was equivalent to the CMC value of the nanomicelle (Zhao et al., 2017; Ding et al., 2018).

#### 2.3.3. Particle size and zeta potential analysis

The particle size and Zeta potential of AMF-loaded TPGS/soluplus mixed micelles were measured by dynamic light scattering technique, and each sample was measured in triplicate. The results were expressed as mean size ± standard deviation (SD) for three separate experiments.

#### 2.3.4. Drug loading and encapsulation efficiency

The encapsulation efficiency (EE) and drug loading (DL) were determined by HPLC. A chromatographic column (ZORBAX SB-C18, 5 μm, 4.6 mm × 150 mm, Agilent, Palo Alto, CA) was used. The mobile phases were water (A, 0.1% of formic acid) and acetonitrile (B), and the flow rate was 1 mL/min. Gradient elution was adopted and the elution procedure was as follows: 35–45% B (0–10 min), 45–35% B (10–10.1 min), and 35% B (10.1–15 min). The sample was injected at a volume of 20 μL and the detection wavelength was 338 nm.

*For the EE determination*: the prepared mixed micelles solution (200 μL) was dissolved in 2 mL methanol, and then treated with ultrasonic for 10 min to release the encapsulated drug. The solution was then centrifuged twice at 21,380×*g* for 10 min. Take the supernatant sample for analysis.

*For the DL determination*: The prepared micelle solution was freeze-dried. And 45 mg of the powder was dissolved in 1 mL water. Subsequently, 200 μL the solution was added to 2 mL methanol, and then treated with ultrasonic for 10 min. The solution was then centrifuged twice at 21,380×*g* for 10 min. Take the supernatant sample for analysis:

$$\text{EE}\%\;={\text{ W}}_{\text{AMF}}/{\text{W}}_{\text{AMF}}’\times \text{100}\%$$

$$\text{DL}\%\;={\;W}_{\text{AMF}}/W_{\text{micelle}}\times 100\%$$

(Zhai et al., 2013; Yan et al., 2016).

W_AMF_ is the weight of drug in micelles; W_AMF_’ is the weight of feeding drug; W_micelle_ is the total weight of feeding soluplus, TPGS, and drug in micelles.

#### 2.3.5. Optimization of preparation conditions

Because the ratio of drugs and excipients has a great influence on the particle size, encapsulation efficiency, and drug loading of the micelle, the ratio of AMF and excipients was optimized in this experiment. Particle size, PDI, Zeta potential, and encapsulation efficiency were used as the indexes. The results are shown in Table 1.

**Table 1.** Characteristics of AMF-loaded TPGS/Soluplus mixed micelles prepared by different ratios of soluplus and TPGS.

AMF:TPGS:soluplus	Size (nm)	PDI	Zeta (mV)	EE (%)
1:10:20	97.72 ± 0.74	0.253 ± 0.001	−1.86 ± 0.26	23.52 ± 0.67
1:10:30	67.33 ± 2.01	0.081 ± 0.021	−0.84 ± 0.04	99.18 ± 0.76
1:10:40	65.35 ± 1.14	0.057 ± 0.032	−0.10 ± 0.12	98.45 ± 0.85
1:10:50	67.80 ± 2.89	0.066 ± 0.025	0.13 ± 0.29	94.15 ± 0.40

#### 2.3.6. Micelle stability

To test the optimal formulation’s storage stability, the AMF-loaded TPGS/soluplus mixed micelles was stored at 4 °C for 60 d. Particle size, PDI, Zeta potential, and encapsulation efficiency of the drug-loaded micelles were measured at 0 d, 15 d, 30 d and 60 d, respectively, and the clarification of the micelles was observed.

#### 2.3.7. Cell culture

A549 cells were used in the cytotoxicity and cellular uptake experiments of the AMF-loaded TPGS/soluplus mixed micelles. A549 cells were cultured in F-12 medium containing 1% penicillin–streptomycin and 10% fetal bovine serum. And the cells were maintained in an incubator at 37 °C in a humidified atmosphere of 5% CO_2_.

#### 2.3.8. *In vitro* cytotoxicity

*In vitro* cytotoxicity of AMF-loaded TPGS/soluplus mixed micelles was determined using the CCK-8 assay. The cells were seeded in 96-well plates at a density of 5000 cells/well, and incubated 24 h to allow cell attachment. After 24 h, the original medium was sucked out. Then, the cells were incubated with AMF and AMF-loaded TPGS/soluplus mixed micelles of different concentration. The concentrations of AMF was ranged from 2000 μg/mL to 15.6 μg/mL, and AMF-loaded TPGS/soluplus mixed micelles ranged from100 μg/mL to 0.78 μg/mL. The cells were incubated for another 24 h. And then the medium was removed, fresh medium and CCK-8 (10 μL) were added and the cells were incubated for 2 h. Finally, the absorbance at 450 nm was measured using a microplate reader, and each treatment was measured for three times (Bernabeu et al., 2016).

#### 2.3.9. *In vitro* cellular uptake

To evaluate the cellular uptake of AMF and AMF-loaded TPGS/soluplus mixed micelles, AMF collected from A549 cell was determined by HPLC. Briefly, A549 cells were seeded in 6-well plates at the density of 5 × 10^5^ cells/well and allowed to attach for 24 h at 37 °C in CO_2_. The original medium was removed, and each well was washed with PBS for three times. Then AMF and AMF-loaded TPGS/soluplus mixed micelles were added at the concentration of 30 μg/mL and the cells were incubated for 1 h, 2 h, 4 h and 6 h, respectively. Untreated cells acted as control. At predetermined time-points, the medium was removed and the cells were rinsed with PBS. Then 0.25 mL trypsin was added to digest the cells. When the cells are digested, they were collected in EP tube and centrifuged at 157 × g for 5 min. The supernatant was removed and the cells were resuspend with 200 μL PBS. The cells were broken with ultrasonic for 5 min and centrifuged at 21,380 × g for 10 min. AMF content in the supernatant was measured by HPLC method and the protein content was also determined using BCA protein assay kit according to the manufacturer’s protocol. All experiments were repeated in triplicate (Bernabeu et al., 2016).

Cell uptake of AMF = Intracellular AMF concentration (μg/mL)/intracellular protein concentration (mg/mL)

In the study, to analyze the results of cellular uptake more comprehensively, a visual qualitative analysis was carried out. The detail method was as follows: A549 cells with good growth status were seeded in 4× culture dishes at 1.5 × 10^5^ cells/well, cultured overnight at 37 °C for 24 h. Then the old medium was removed and washed with PBS for three times. Add the prepared culture medium with the drug concentration at 30 μg/mL, and continue to culture at 37 °C for 1 h, 2 h, 4 h and 6 h. The fluorescence intensity of cells was observed under the laser confocal microscope at different time points. The intensity of fluorescence represented the amount of drug intake.

#### 2.3.10. Metabolite analysis of AMF and AMF-loaded TPGS/soluplus mixed micelles

##### 2.3.10.1. Instrumentation and conditions

UHPLC was performed on Shimazu’s ultra-high performance liquid chromatography system (Kyoto, Japan), which was equipped with a triple TOF™ 5600^+^ MS/MS system (AB SCIEX, Redwood City, CA). The chromatographic separation was carried on Poroshell 120 EC-C18 (2.1 × 100 mm, 2.7 μm) with a Security Guard® UHPLC C_18_ pre-column (Poroshell). The mobile phases were water (A, 0.1% formic acid) and acetonitrile (B), and the elution procedures were as follows: 2 0–36% B (0–5 min), 3 6–59% B (5–14 min), 59–73% B (1 4–18 min), 73–95%B (18–21 min), and 95% B (21–25 min). The flow rate was 0.3 mL/min and the injection volume was 2 μL. The column temperature was 40 °C, and the automatic injector temperature was 4 °C.

A Triple TOF™ 5600 system with Duo-Spray™ ion sources operating in the negative electrospray ionization mode was used for the detection. The optimized conditions of mass spectrometry as follows: ion source voltage −4.5 kV; ion source temperature, 550 °C; declustering potential (DP), 60 V; collision energy (CE), −40 V + 15 eV. Atomization gas (Gas 1, N2), heat gas (Gas 2, N2), and curtain gas pressure was 55 psi, 55 psi, and 35 psi, respectively. The parent ion and the daughter ion scanning range were 50–800 Da and 50–1000 Da, respectively. Data were collected in IDA mode, and an automated calibration delivery system (CDS) was used to calibrate the MS and MS/MS automatically. The data were collected using the Analyst TF 1.6.1 software for 25 min.

##### 2.3.10.2. Animals and drug administration

Sprague–Dawley (SD) male rats (Certificate NO. 181 1164, 200 ± 20 g) were provided by the experimental animal center of Hebei Medical University. All animal experiments followed the guidelines of the experimental animal management committee of Hebei Medical University. The rats were housed under standard conditions (temperature, 22–24 °C, relative humidity, 45–55%, and light, 12 h dark 12 light cycles) for 5 d prior to use, and fasted 12 h before experiment, but free to water.

The experimental rats were randomly divided into 12 groups with 3 rats per group (groups 1 and 2, the blank blood sample groups; group 3 and 4, the blank bile sample groups; groups 5 and 6, the blank urine and feces sample groups; group 7 and 8, the experimental blood sample groups; groups 9 and 10, the experimental bile sample groups; group 11 and 12, the experimental urine and feces sample groups). The volume of gavage was 16 mL/kg. The prepared AMF suspension was orally administered to 9 rats of groups 7, 9 and 11 at a dose of 100 mg/kg, and the prepared AMF-loaded TPGS/soluplus mixed micelles was orally administered to 9 rats of groups 8, 10, and 12 at a dose of 100 mg/kg (equal to the AMF). Groups 1, 3, and 5 were given aqueous solution of 0.5% CMC-Na, and groups 2, 4, and 6 were given blank micelle of the same concentration as the IGG-loaded micelle.

##### 2.3.10.3. Bio-sample collection

*Plasma samples*: Approximately 300–500 μL blood was collected from the canthus of each rat at 5 min, 10 min, 15 min, 30 min, 1 h, 2 h, 3 h, 6 h, 9 h, 12 h, and 24 h after gavage. The blood samples were centrifuged at 1920×*g* for 10 min to obtain the plasma, and then the samples were merged.

*Bile samples*: After intragastric administration, each rat was subcutaneously injected with urethane solution at a dose of 1.0 g/kg. After anesthesia, the bile duct was intubated and the bile was collected for 24 h.

*Feces and urine samples*: The rats were put into a metabolic cage after administration, fasted, but can drink freely. The urine and feces samples were collected at regular intervals for 72 h, and the urine and feces were merged, respectively.

##### 2.3.10.4. Bio-sample pretreatment

For plasma, bile and urine pretreatment, an aliquot of 2 mL bio-sample was vortexed with 6 mL of methanol for 5 min and centrifuged for 10 min at 4 °C and 21,380×*g*. The supernatant was taken and dried under nitrogen gas at room temperature.

For feces, 6 mL methanol was added to 1 g feces, ultrasonically extracted for 30 min at 25 °C after vortexed, centrifuged at 21,380×*g* for 10 min, and the supernatant was taken. Repeat the above process once, and the supernatant was merged. The supernatants were evaporated to dryness under nitrogen gas.

Before analysis, the residue was dissolved with 100 mL methanol, vortexed and centrifuged twice at 21,380×*g* for 10 min. The supernatant was injected for analysis.

## 3. Results

### 3.1. Determination of CMC

**Figure 1.**
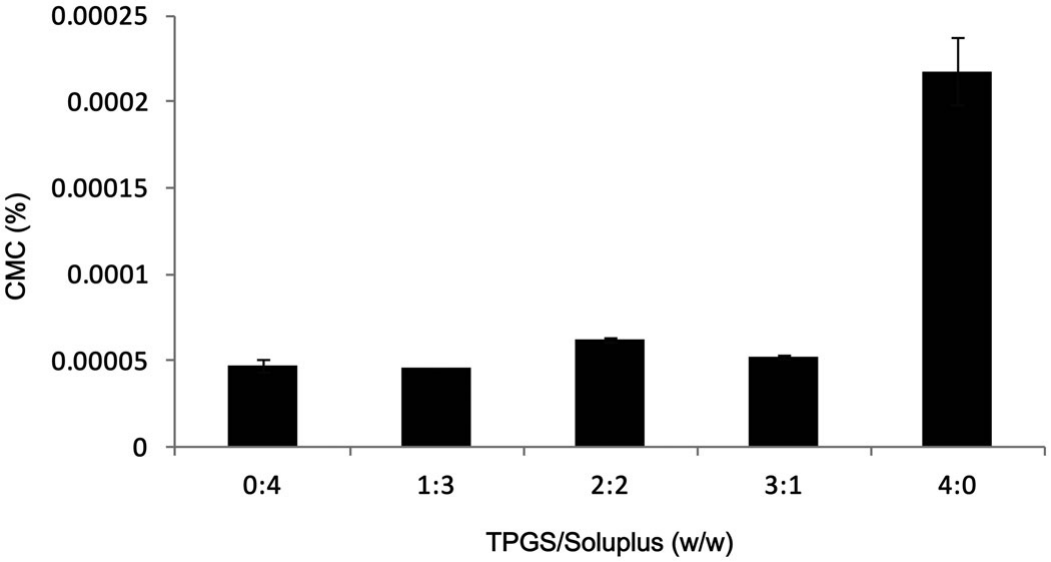
CMC values for AMF-loaded TPGS/soluplus mixed micelles.

The CMC was used as an important indicator to evaluate the stability of micelles. In this experiment, the iodine hydrophobic probe method was used to determine the CMC. As a small hydrophobic molecule, the soluble iodine preferred located in the hydrophobic microenvironment of the copolymer, leading to the conversion of I^-^ to I_2_ in the solution. Therefore, the ultraviolet absorption of the solution changed, and it can be measured with an ultraviolet spectrophotometer. The measured mass ratios of TPGS and soluplus were 0: 4, 1: 3, 2: 2, 3: 1 and 4: 0. Their CMC values are shown in Figure 1. It can be seen from the figure that the CMC values of different mass ratios were very low. When the mass ratio of TPGS and soluplus was 1: 3, the CMC value of the micelles was the lowest. Due to its low CMC, mixed micelles have high stability and the ability to maintain their integrity even when diluted in the blood circulation was relatively insensitive, and have a longer circulation time compared to surfactant micelles *in vivo* (Oerlemans et al., 2010).

### 3.2. Optimization of preparation conditions

It can be seen from the CMC results that when the mass ratio of TPGS to soluplus was 1:3, the CMC value was the lowest, indicating that the micelle was the most stable. And it can be seen from Figure 1 that when the proportion of soluplus was high, the CMC value of micelle was generally low. Therefore, according to previous experience, the mass ratio of TPGS and soluplus was optimized in this experiment, especially the amount of soluplus. The result is shown in Table 1. When the ratio of AMF, TPGS, and soluplus was 1:10:30, 1:10:40, and 1:10:50, all the indexes were ideal. When the mass ratio was 1:10:30, encapsulation efficiency of the micelle was the highest and the consumption of auxiliary materials was relatively minimal. Therefore, in the principle of saving reagents and cost, the final ratio of 1:10:30 was selected for micelle preparation.

### 3.3. Determination of particle size, Zeta potential, encapsulation efficiency and drug loading

**Figure 2.**
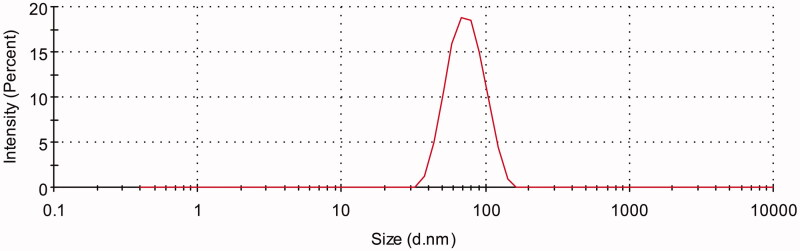
Size distribution of AMF-loaded TPGS/soluplus mixed micelles measured by dynamic light scattering.

Three batches of AMF-loaded TPGS/soluplus micelles were prepared according to the ratio of 1:10:30 (AMF:TPGS:soluplus). Figure 2 shows that the nanomicelles had an average particle size of 67.33 ± 2.01 nm with a PDI of 0.080667 ± 0.021008. The surface charge of AMF-loaded mixed micelles was neutral with zeta potential of 0.84133 ± 0.041405 mV. And the encapsulation efficiency and drug loading measured by HPLC were 99.18 ± 0.76% and 2.47 ± 0.01%, respectively.

### 3.4. Micelle stability

**Figure 3.**
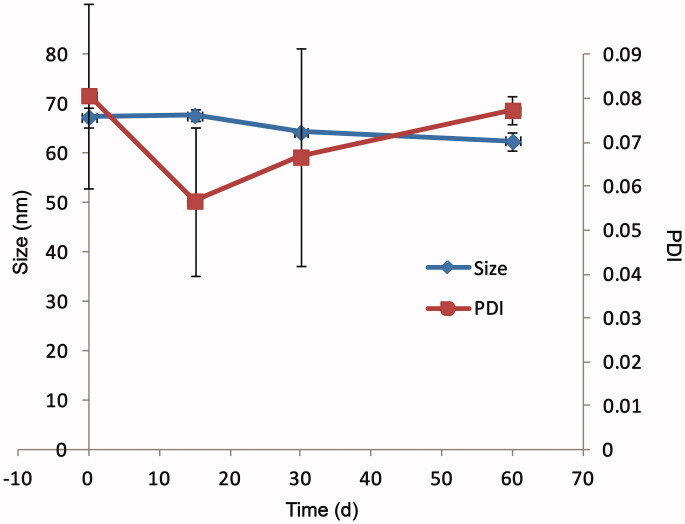
The particle size and PDI of AMF-loaded mixed micelles at 4 °C after storage for 60 d.

The AMF-loaded mixed micelles was stored at 4 °C for 60 d. Particle size, PDI, Zeta potential, and encapsulation efficiency of micelle were determined and the clarity of the micelles was observed at 0 d, 15 d, 30 d, and 60 d. The results are shown in Figure 3 and Table 2. It could be seen from Figure 3 that there was no trend of polymerization between micelles, and Zeta potential did not change significantly. And the results of encapsulation rate indicated that it was difficult for micelles to self-degrade. From the appearance, micelle solution was still transparent and clear, without precipitation. The above results indicated that the AMF micelles could be kept relatively stable at 4 °C for a certain time.

**Table 2.** Characteristics of AMF-loaded TPGS/soluplus mixed micelles stored for 0 d, 15 d, 30 d and 60 d.

	0 d	15 d	30 d	60 d
Size (nm)	67.33 ± 2.01	67.71 ± 1.06	64.28 ± 0.64	62.24 ± 1.76
PDI	0.081 ± 0.021	0.057 ± 0.017	0.067 ± 0.025	0.077 ± 0.003
Zeta potential (mV)	−0.84 ± 0.04	−0.17 ± 0.24	−0.40 ± 0.20	−0.19 ± 0.13
EE (%)	99.18 ± 0.76	98.96 ± 0.34	98.69 ± 0.40	98.52 ± 0.40
Clarity	Clear	Clear	Clear	Clear

### 3.5. *In vitro* cytotoxicity

**Figure 4.**
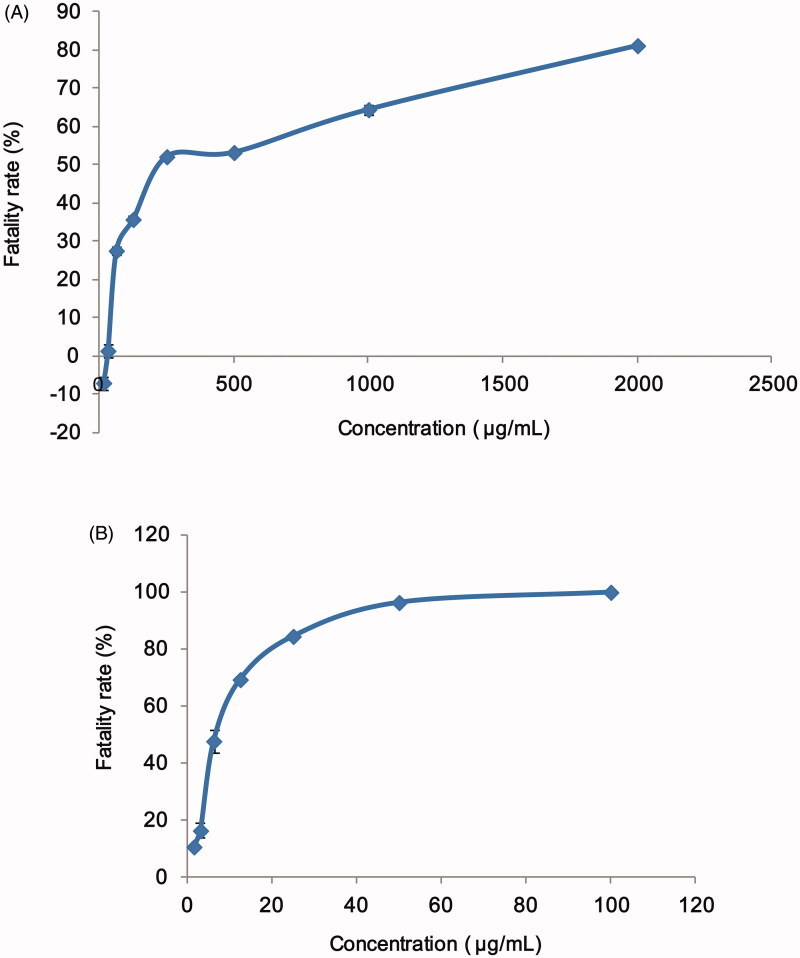
Fatality rate of A549 cell after 24 h of treatment with (A) AMF and (B) AMF-loaded TPGS/soluplus mixed micelles.

A549 cells were commonly used in the study of cytotoxicity. In this study, the cell fatality rates were measured after incubated in different concentrations of AMF and AMF-loaded mixed micelles. The results are shown in Figure 4. And the IC_50_ value of AMF and AMF-loaded mixed micelles calculated by GraphPad Prism 5 (GraphPad Software, La Jolla, CA) were 83.09 ± 0.65 μg/mL and 6.11 ± 0.74 μg/mL, respectively. As can be seen from the figure and the calculation results, the IC_50_ value of AMF-loaded micelles was almost one-twelfth of that of AMF, indicating that AMF-loaded micelles have much greater toxicity to A549 cells than AMF. Therefore, it was speculated that AMF-loaded micelles had much higher lethality to cancer cells than AMF, and had the potential to treat cancer. At the same time, it can be seen that when AMF was at a lower concentration, the fatality rate of A549 cells was less than 0, which indicating that the low concentration of AMF had a small inhibitory effect on A549 cells, and the cells continued growing under conditions of sufficient nutrition, so the cell concentration showed an increasing trend, and the cell inhibition rate was less than 0.

### 3.6. *In vitro* cellular uptake

**Figure 5.**
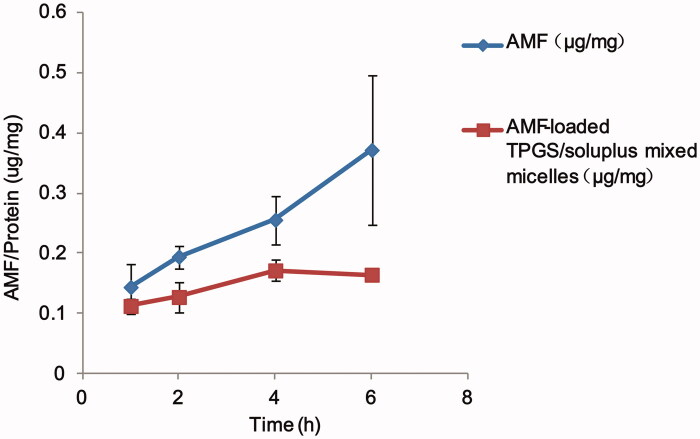
Time-dependent intracellular uptake in A549 cell lines for AMF and AMF-loaded TPGS/soluplus mixed micelles. Drug amount was normalized by protein concentrations of the cell lysates. Results are expressed as mean ± S.D. (*n* = 3).

The results of cellular uptake of AMF monomers and AMF-loaded TPGS/soluplus mixed micelles are shown in Figure 5. Figure 5 indicates that the cell uptake of AMF-loaded mixed micelles was a time and concentration-dependent process. The cellular uptake of AMF monomers increased obviously with the extension of time in 1–6 h, and the cellular uptake of AMF-loaded TPGS/soluplus mixed micelles increased in 1–4 h, and remained almost unchanged in 4–6 h. Moreover, the cellular uptake of AMF-loaded TPGS/soluplus mixed micelles was significantly lower than that of AMF (*p* < .05), which was due to the negative charge on the micelle and the negative charge on the outer surface of the cell membrane, resulting in a lower uptake of the micelle than that of the monomer.

**Figure 6.**
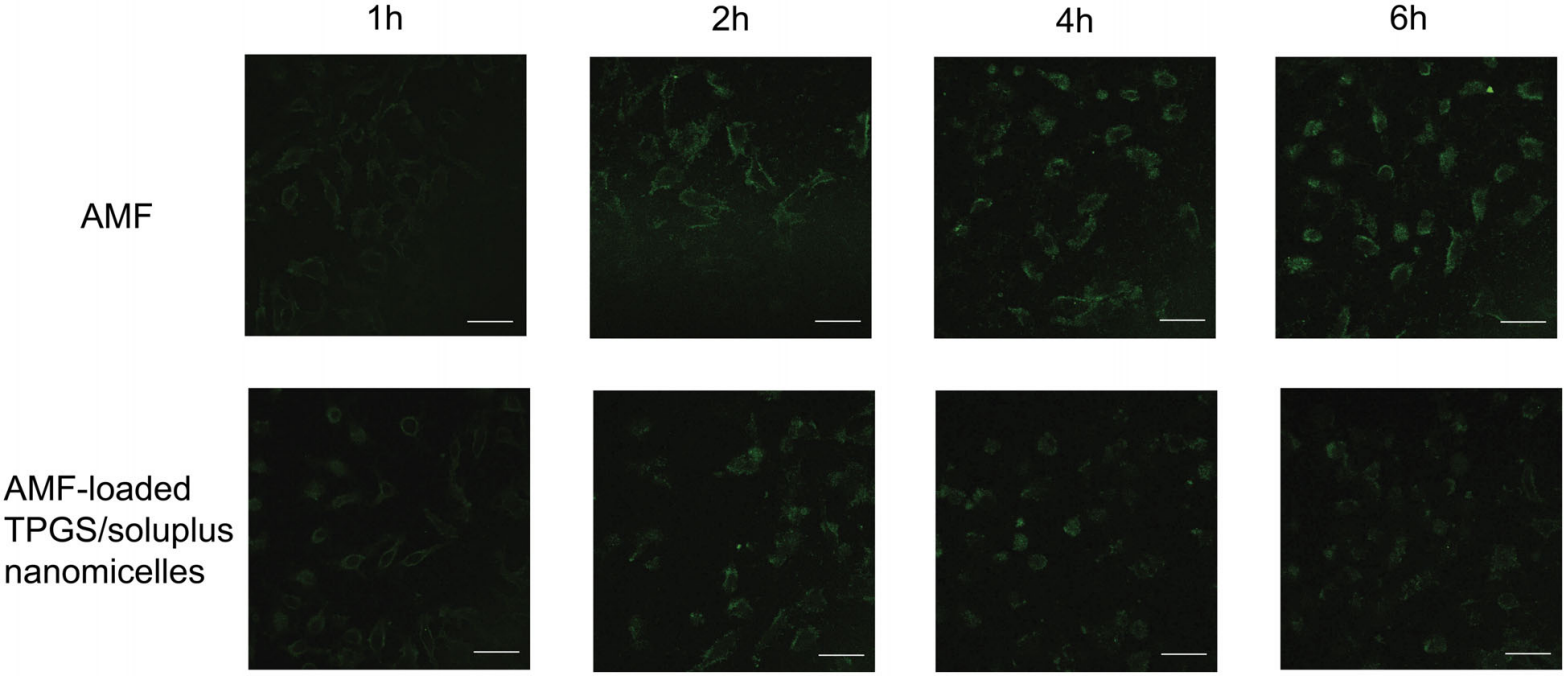
Cellular uptake of AMF and AMF-loaded TPGS/soluplus mixed micelles in A549 cells observed by laser confocal microscope. Scale bar is 50 μm.

As shown in Figure 6, it could be seen that the cellular uptake of AMF and AMF-loaded TPGS/soluplus mixed micelles had a clear trend of increase over time. And the cellular uptake of AMF was higher than that of mixed nanomicelles, which was consistent with our previous results. From Figure 6, it can also be seen that in the samples incubated with AMF, the morphology of the cells did not change significantly with the extension of time, and the cells had not been lysed. But in the mixed nanomicelle incubated samples, the drug gradually entered the cells with the prolongation of time, and the cells tended to die. By 6 h, it can be seen that the cells had obvious lysis and the drug was gradually released. The result also suggested that under the same drug concentration, the cytotoxicity of AMF was significantly lower than that of AMF-loaded TPGS/soluplus mixed micelles.

### 3.7. Analysis of the metabolites of AMF and AMF-loaded TPGS/soluplus mixed micelles

#### 3.7.1. Metabolites and metabolic pathway of AMF

**Figure 7.**
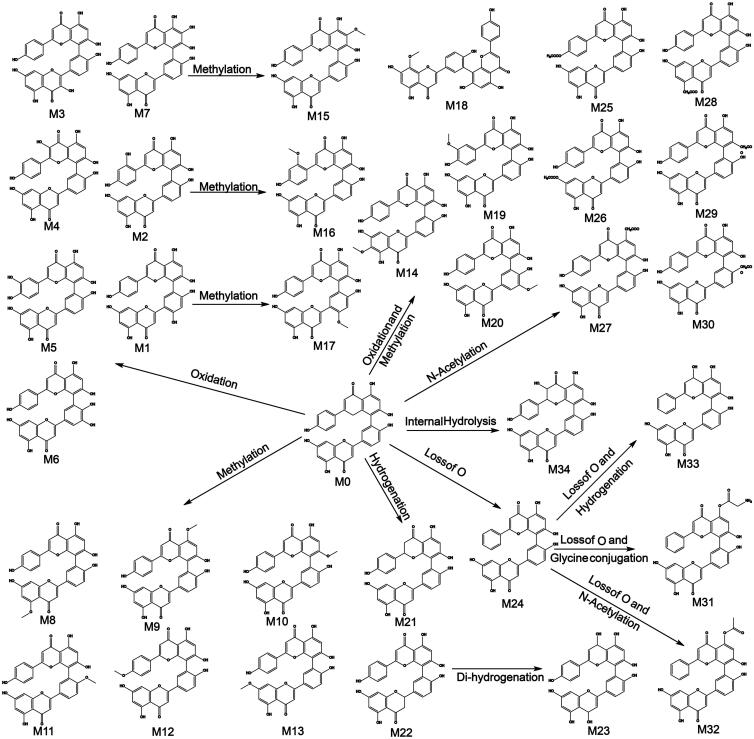
Metabolic profile and proposed metabolic pathways of AMF in rats.

**Figure 8.**
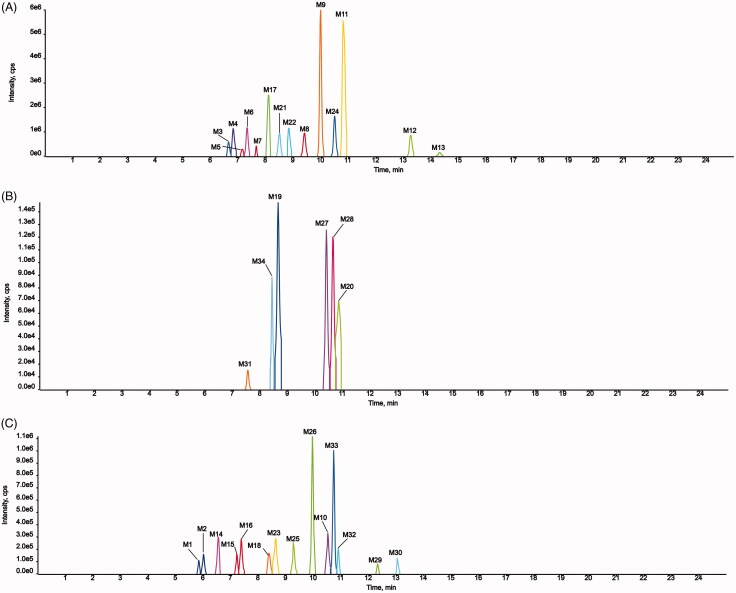
Extracted ion chromatograms of all metabolites of AMF in rats.

In this study, a total of 34 metabolites of AMF were found. Metabolites were mainly distributed in feces, including 34 metabolites (M1–M34), and 3 metabolites were in urine (M4, M7, and M17). No metabolite of AMF was found in bile or plasma. The result was shown in Figures 7 and 8 and Table 3.

**Table 3.** Summary of metabolites of AMF in rats.

Metabolite ID	Name	Formula	*m*/*z*	ppm	R.T. (min)	MS/MS Fragments	Clog P	Plasma	Bile	Urine	Feces
M1	Oxidation	C_30_H_18_O_11_	553.0748	−5.2	5.80	535.3241, 399.0500, 375.0502, 133.0282	3.99244	−	−	−	+
M2	Oxidation	C_30_H_18_O_11_	553.0746	−5.4	6.04	493.2843, 375.2439, 159.0452, 117.0346	3.99244	−	−	−	+
M3	Oxidation	C_30_H_18_O_11_	553.0749	−5.0	6.63	443.0731, 375.0507, 159.0441, 117.0345	4.15118	−	−	−	+
M4	Oxidation	C_30_H_18_O_11_	553.0761	−2.7	6.80	443.0384, 375.0494, 331.0590, 307.0583	4.15118	−	−	+	+
M5	Oxidation	C_30_H_18_O_11_	553.0749	−4.9	7.11	443.0385, 375.0501, 133.0284, 117.0344	4.36244	−	−	−	+
M6	Oxidation	C_30_H_18_O_11_	553.0754	−4.0	7.30	417.0234, 159.0440, 117.0388, 133.0316	4.36244	−	−	−	+
M7	Oxidation	C_30_H_18_O_11_	553.0750	−5.4	7.65	467.0776, 443.0438, 375.0504, 117.0130	4.38868	−	−	+	+
M8	Methylation	C_31_H_20_O_10_	551.0967	−3.0	9.39	493.0529, 417.0597, 399.0490, 375.0376	4.64265	−	−	−	+
M9	Methylation	C_31_H_20_O_10_	551.0975	−1.5	9.99	551.0975, 431.0744, 413.0631, 389.0634	4.64643	−	−	−	+
M10	Methylation	C_31_H_20_O_10_	551.0952	−5.7	10.51	457.0517, 431.0749, 375.0493, 331.0602	5.28643	−	−	−	+
M11	Methylation	C_31_H_20_O_10_	551.0978	−1.1	10.82	457.0529, 431.0742, 375.0488, 159.0431	5.29394	−	−	−	+
M12	Methylation	C_31_H_20_O_10_	551.0969	−2.7	13.25	483.1061, 399.0832, 321.0377, 283.0233	5.53754	−	−	−	+
M13	Methylation	C_31_H_20_O_10_	551.0974	−1.7	14.31	507.1041, 413.0503, 389/0608, 375.0588	5.54265	−	−	−	+
M14	Oxidation and methylation	C_31_H_20_O_11_	567.0904	−5.0	6.53	455.0397, 417.0597, 387.0479, 331.0578	4.46777	−	−	−	+
M15	Oxidation and methylation	C_31_H_20_O_11_	567.0908	−4.3	7.21	499.3021, 387.0500, 233.0441, 141.0680	4.4689	−	−	−	+
M16	Oxidation and methylation	C_31_H_20_O_11_	567.0905	−4.8	7.36	549.0821, 499.2736, 387.0500, 189.0547	4.49093	−	−	−	+
M17	Oxidation and methylation	C_31_H_20_O_11_	567.0917	−2.8	8.08	447.0683, 405.0583, 375.0490, 147.0438	4.49585	−	−	+	+
M18	Oxidation and methylation	C_31_H_20_O_11_	567.0909	−4.2	8.37	451.0231, 433.0092, 409.0112, 117.0337	4.71777	−	−	−	+
M19	Oxidation and methylation	C_31_H_20_O_11_	567.0909	−4.2	8.64	499.3074, 375.0506, 351.0500, 309.0400	4.80093	−	−	−	+
M20	Oxidation and methylation	C_31_H_20_O_11_	567.0929	−1.5	10.83	473.0607, 447.0701, 405.0592, 375.0496	4.80585	−	−	−	+
M21	Hydrogenation	C_30_H_20_O_10_	539.0958	−4.8	8.47	445.0451, 413.0654, 135.0439, 117.0339	3.16655	−	−	−	+
M22	Hydrogenation	C_30_H_20_O_10_	539.0960	−4.4	8.81	539.0594, 375.0487, 309.0386, 119.0493	3.16769	−	−	−	+
M23	Di-hydrogenation	C_30_H_22_O_10_	541.1117	−4.4	8.62	497.1198, 421.0607, 353.0641, 161.0603	1.37976	−	−	−	+
M24	Loss of O	C_30_H_18_O_9_	521.0862	−3.0	10.49	503.3300, 399.0482, 375.0492	5.61404	−	−	−	+
M25	N-Acetylation	C_32_H_20_O_11_	579.0909	−4.1	9.27	537.0810, 485.0482, 493.0903, 375.0493	4.96754	−	−	−	+
M26	N-Acetylation	C_32_H_20_O_11_	579.0915	−3.0	9.95	561.0807, 537.0805, 375.0494	4.97265	−	−	−	+
M27	N-Acetylation	C_32_H_20_O_11_	579.0902	−5.4	10.39	561.3370, 537.0823, 417.0605, 375.0501	5.12559	−	−	−	+
M28	N-Acetylation	C_32_H_20_O_11_	579.0909	−4.1	10.63	561.3431, 443.0397, 399.0492, 375.0498	5.12559	−	−	−	+
M29	N-Acetylation	C_32_H_20_O_11_	579.0922	−1.9	12.31	459.0693, 417.0626, 375.0584	6.02559	−	−	−	+
M30	N-Acetylation	C_32_H_20_O_11_	579.0922	−1.9	13.04	459.0725, 417.0626, 399.0499, 349.0719	6.11304	−	−	−	+
M31	Loss of O and glycine conjugation	C_32_H_21_NO_10_	578.1060	−5.7	7.53	417.0617, 375.0503, 306.1181, 150.0375	3.71729	−	−	−	+
M32	Loss of O and N-Acetylation	C_32_H_20_O_10_	563.0960	−4.3	10.88	545.0876, 357.0747, 383.0528	4.73389	−	−	−	+
M33	Loss of O and hydrogenation	C_30_H_20_O_9_	523.1013	−4.1	10.72	417.0596, 375.0485, 307.0589	3.83469	−	−	−	+
M34	Internal hydrolysis	C_30_H_20_O_11_	555.0918	−2.7	8.44	509.2884, 487.3033, 403.0834	3.41608	−	−	−	+

+: Detected; −: undetected.

In the metabolism, metabolites were mainly distributed in feces, and none of them were found in bile or plasma, which indicated that the bioavailability of AMF was low. The main metabolic pathways were oxidation, methylation, oxidation and methylation, acetylation, hydrogenation. Oxidation is its main metabolic reaction, which is related to its antioxidant properties. The results are shown in Figure 7. No monoflavone was found in the metabolites of AMF, which indicated that the biflavones formed by C–C bonding had stable structure and were not easy to break into monoflavone, which was different from that conjugated by ether bond (Chen et al., 2019).

#### 3.7.2. Metabolite analysis and metabolic pathway of AMF-loaded TPGS/soluplus mixed micelles

**Figure 9.**
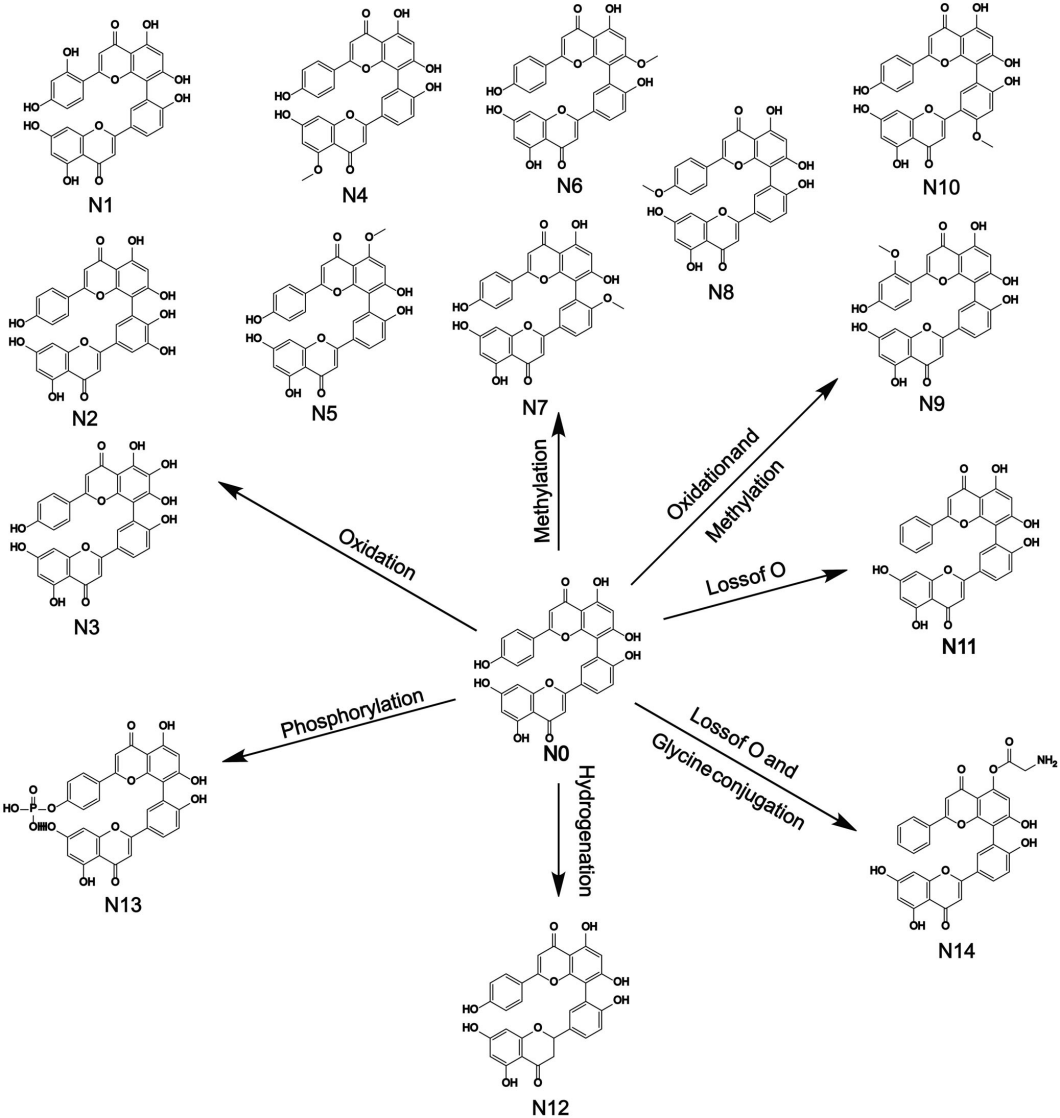
Metabolic profile and proposed metabolic pathways of AMF-loaded TPGS/soluplus mixed micelles in rats.

**Figure 10.**
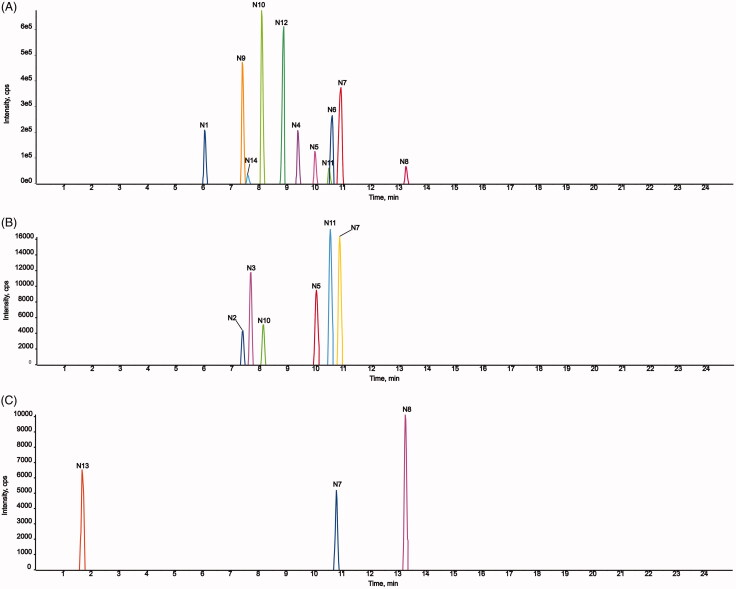
Extracted ion chromatograms of all metabolites of AMF-loaded TPGS/soluplus mixed micelles in rats (A, in rat feces; B, in rat urine; C, in rat plasma).

Fourteen metabolites of AMF-loaded micelles were found in rats, including three in plasma (N7, N8, and N13), six in urine (N2, N3, N5, N7, N10, and N11), and 11 in feces (N1, N4-N12, and N14). No metabolites were found in bile, as shown in Figures 9 and 10 and Table 4.

**Table 4.** Summary of metabolites of AMF-loaded TPGS/soluplus mixed micelles in rats.

Metabolite ID	Name	Formula	*m*/*z*	ppm	R.T. (min)	MS/MS fragments	Clog P	Plasma	Bile	Urine	Feces
N1	Oxidation	C_30_H_18_O_11_	553.0774	−0.4	6.04	485.0855, 375.0505, 331.0602, 133.0285	3.99244	−	−	−	+
N2	Oxidation	C_30_H_18_O_11_	553.0774	−0.5	7.30	433.0500, 391.0488, 333.0409, 174.9532	4.36244	−	−	+	−
N3	Oxidation	C_30_H_18_O_11_	553.0777	0.1	7.65	417.0641, 375.0511, 347.0530, 117.0332	4.38868	−	−	+	−
N4	Methylation	C_31_H_20_O_10_	551.0980	−0.6	9.39	519.0735, 431.0764, 389.0635, 375.0494,	4.64265	−	−	−	+
N5	Methylation	C_31_H_20_O_10_	551.0986	0.5	9.99	533.3134, 483.0851, 389.0677, 375.0522	4.64643	−	−	+	+
N6	Methylation	C_31_H_20_O_10_	551.0976	−1.5	10.51	483.1010, 389.0667, 190.9966	5.28643	−	−	−	+
N7	Methylation	C_31_H_20_O_10_	551.0980	−0.7	10.82	483.0173, 389.0686, 345.0753	5.29394	+	−	+	+
N8	Methylation	C_31_H_20_O_10_	551.0985	0.1	13.25	519.0701, 483.1087, 283.0241	5.53754	+	−	−	+
N9	Oxidation and methylation	C_31_H_20_O_11_	567.0934	0.2	7.36	417.0620, 375.0516, 331.0614, 189.0559	4.49093	−	−	−	+
N10	Oxidation and methylation	C_31_H_20_O_11_	567.0935	0.4	8.08	417.0601, 375.0504, 331.0607, 189.0533	4.49585	−	−	+	+
N11	Loss of O	C_30_H_18_O_9_	521.0882	0.7	10.49	503.3361, 399.0497, 375.0523	5.52659	−	−	+	+
N12	Hydrogenation	C_30_H_20_O_10_	539.0985	0.3	8.81	419.0536, 309.0391, 119.0484	3.16769	−	−	−	+
N13	Phosphorylation	C_30_H_19_O_13_P	617.0467	−3.8	1.67	446.9946, 423.1064, 397.0020	2.97424	+	−	−	−
N14	Loss of O and glycine conjugation	C_32_H_21_NO_10_	578.1092	0	7.53	417.0627, 399.0467, 375.0509, 331.0601	3.71729	−	−	−	+

+: Detected; −: undetected.

Among the metabolites, five were phase I metabolites and nine were phase II metabolites. The main metabolic pathways were oxidation, methylation, oxidation and methylation, loss of O, hydrogenation, loss of O and glycine conjugation and phosphorylation. Methylation and oxidation were the main metabolic pathways. The results are shown in Figure 9.

## 4. Discussion

In the determination of encapsulation efficiency of AMF-loaded micelles, the micelles were first diluted with methanol, then sonicated in water-bath, filtered by 0.22 μm nylon filter membrane, and injected for analysis. During the process, it was found that the encapsulation efficiency of micelles prepared by the same ratio and method was unstable, and the encapsulation efficiency of micelles prepared by different ratios was low. Because the method of micelles preparation was the same, it was speculated that nylon filter membranes had the sorption at different degrees to AMF. Therefore, after demulsification, the sample was centrifuged twice but not filtered.

The CMC of different carrier ratios measured in the experiment were all low, and when the TPGS/soluplus was 1: 3, the CMC was the lowest, indicating that the mixed nanomicelles prepared at this ratio had good stability. AMF-loaded TPGS/soluplus mixed micelles exhibited good stability, which was determined by the structure of micelles. AMF could be wrapped by the two carriers in the core of the nanomicelles, which avoided the direct contact with the matrix and prevented the degradation of drugs by gastric acid and P450 enzyme. As a result, the drug could get into the blood to cycle in the form of micelles, and also delayed the release of drugs, to ensure that it can play a more effective pharmacological role (Torchilin, 2006; Xu et al., 2016). The particle size of the mixed nanomicelles prepared in this experiment was 50–100 nm, which was small enough. Compared with large-size drugs, it can promote the absorption of poorly soluble drugs, significantly improve the absorption of drugs in the gastrointestinal tract, and reduce filtration of glomeruli to improve drug bioavailability (Wu et al., 2019).

In this study, AMF-loaded TPGS/soluplus mixed micelles showed higher cytotoxicity, which may be related to the toxicity of drug carrier TPGS to cancer cells. The anticancer activity of TPGS was reported to be associated to its apoptosis inducing properties by the generation of reactive oxygen species, which might be the reason for the toxicity of high concentration of blank micelles to cancer cells. It has been reported that the toxicity of soluplus-encapsulated drugs to cancer cells was significantly lower than that of TPGS/soluplus-encapsulated mixed nanomicelles (Bernabeu et al., 2016), which suggested that TPGS played an important role in increasing drug toxicity. The cytotoxicity of the mixed nanomicelles was high, which may be related to the combined effect of carrier and drug encapsulation (Bernabeu et al., 2016; Ding et al., 2018).

In the experiments of cytotoxicity and cellular uptake, we found that the IC_50_ value of AMF-loaded mixed micelles was much lower than that of AMF monomers, but its cellular uptake was significantly lower than that of AMF. This result could be related with the cell response (motility increment) to the antineoplastic drug over the first 6 h of exposure (Cagel et al., 2017). Or it may be related to the inhibition of some extracellular signaling pathways, such as NF-kappa B/MAPKs signaling pathway and Hedgehog/Glil signaling pathway (Zhang et al., 2018; Bao et al., 2019), which resulted in the abnormal growth and differentiation of cells, thus causing cell death. Under laser confocal microscopy, it can be seen that after 4 h, the cell morphology incubated with mixed nanomicelles began to change significantly, and the change in cell viability affected the absorptive capacity of cells, resulting in less cellular uptake of mixed nanomicelles. In this case, the data confirmed that the novel AMF-loaded mixed micelles we prepared can have a toxic effect on cancer cells in a different way compared with most of the micelles (Bernabeu et al., 2016; Cagel et al., 2017; Hu et al., 2017; Ding et al., 2018; Gileva et al., 2019; Shishir et al., 2019). The factors such as cell type and density, particle size, surface modifying ligands, and surface properties can also affect cellular uptake (Wu et al., 2019).

By comparing the metabolites of AMF monomers with those of AMF-loaded micelles in rats, it was found that the metabolites of AMF monomers were mainly distributed in feces, 34 metabolites were found, only 3 metabolites were found in urine, and no metabolites were found in plasma and bile, which indicated that AMF monomers were difficult to enter blood, urine and bile. And most drugs passed through stomach, after intestinal metabolism, it was excreted *in vitro* through feces, which proved once again that AMF monomers were water-insoluble and ester-insoluble compounds with very low bioavailability. Metabolites in micelles are also mainly distributed in feces, with 11 metabolites and no metabolites found in bile. However, three metabolites were detected in plasma and six in urine. This indicated that AMF could be absorbed into blood and urine or be absorbed more into the blood when it was prepared into micelles, and the metabolites could be detected. The wider distribution of metabolites indicated that more drugs can enter the blood circulation. Especially in plasma, there was a significant increase in metabolites, which reflected indirectly that the bioavailability of AMF was improved. However, the metabolites of micelles in feces were much less than those of monomers in feces, which illuminated that after encapsulated in micelles, it was difficult for the AMF monomers to be metabolized by enzymes and intestinal flora in the gastrointestinal tract, and they were directly excreted in the form of micelles, which also reflected that the stability of AMF-loaded micelles was good *in vivo*. In the metabolite analysis, not only a variety of AMF metabolites were identified but also the indirect response to the changes in bioavailability, which was killing two birds with one stone.

In this experiment, AMF-loaded TPGS/soluplus mixed nanomicelle with good solubility and stability, high encapsulation efficiency and drug loading, and a particle size of less than 100 nm, which was good for oral absorption, highly toxic to cancer cells and released slowly in the body. It can provide a good choice for the development of anti-cancer drugs.

## 5. Conclusion

The solubility and the stability of AMF were poor. And literatures have reported that the bioavailability of AMF was very low *in vivo*. So in this experiment, the AMF-loaded TPGS/soluplus mixed micelles were prepared, and the properties of the micelles *in vivo* and *in vitro* were evaluated. The size of micelle was small and its physicochemical properties remained relatively stable within 60 d. In the cytotoxicity test, the IC_50_ value of micelles to A549 cells was much smaller than that of monomers, which was about one-twelfth of that of monomers. And the cellular uptake was lower than that of AMF monomers due to the negative electricity on the surface of micelles and cell membranes. The metabolites of AMF monomer and AMF-loaded mixed micelles were also studied. A total of 34 metabolites of AMF monomer were detected. The main metabolic pathways were oxidation, oxidation and methylation, methylation and acetylation. In rats, AMF was metabolized mainly through the gastrointestinal tract and then discharged from the body. It was difficult to be absorbed into the blood and exert its pharmacological effect and its bioavailability was low. Nevertheless, AMF-loaded mixed micelles had 14 metabolites in rats, 11 in feces, 6 in urine, and 3 in plasma. The increase of metabolites in plasma and urine indicated that the bioavailability of AMF was improved. But it was difficult for AMF to be metabolized by intestinal flora and various enzymes after being encapsulated in micelles.

## Supplementary Material

Supplemental Material
