# Supplementary material for: Preparation, evaluation and metabolites study in rats of novel amentoflavone-loaded TPGS/soluplus mixed nanomicelles
Source: Drug Deliv. 2020 Jan 8;27(1):137–50. doi: 10.1080/10717544.2019.1709920 (PMC6968485; doi:10.1080/10717544.2019.1709920)
Supplement: Supplemental Material [file IDRD_A_1709920_SM4788.docx]

**Analysis of the metabolites of AMF and AMF-loaded TPGS/soluplus mixed micelles**

1. ***The strategies of metabolite analysis***

The strategy of metabolite identification was mainly divided into four steps. Firstly, the samples were separated by ultra-high performance liquid chromatography (UHPLC) and then entered into the mass spectrometry data acquisition system. The metabolites of AMF were monitored by full scanning mode, multiple mass defect filter (MMDF) and dynamic background subtraction (DBS). Secondly, the data collected by UHPLC-Q-TOF-MS/MS was imported into Metabolite Pilot^TM^2.0 to screen potential metabolites by means of mass defect filter (MDF), product ion filter (PIF), neutral loss filter (NLF) and other tools. Thirdly, according to the mass spectrometry, and the parent drug cracking rule, the metabolites’ structures given by the Metabolite Pilot^TM^2.0 were inferred and verified. For isomers, metabolites were distinguished by ClogP value calculated by ChemDraw 14.0. In a general way, in the reversed-phase chromatography system, the larger the ClogP value, the smaller the polarity of the compound and the later the retention time. Finally, Master View^TM^ 1.1 software was used to remove metabolites that were also presented in blank samples, and the remaining metabolites were the final metabolites.

1. ***The mass fragmentation behavior of AMF***

AMF was formed by the polymerization of two monoflavones through the C-C bond, which can also be regarded as the dimer of apigenin. The molecular formula of AMF was C_30_H_18_O_10_ and the structure was shown in Fig.1. In order to infer the structure of metabolites more accurately, the MS/MS fragmentation behavior of AMF was investigated in negative ion mode, and the results were shown in the Fig.6 . Under the above conditions of liquid chromatography, AMF was eluted at 6.9 min with the deprotonated molecule ion[M−H]^−^ at *m/z* 537.0830. The fragment ions mainly appeared at *m/z* 519.0721, 493.0915, 451.0809, 443.0390, 417.0597, 399.0490, 375.0496, 357.0376, 331.0590, 307.0585, 203.0328, 159.0433 and 117.0332. Fragment ion at *m/z* 519.0721 was formed by dehydration between hydroxyl in the position of C-4’ of ringⅠ-B and hydroxyl in the position of C-7 of ringⅡ-A. The fragment ion *m/z* 493.0915 was formed by the loss of O and CO. Two γH in the ringⅠ-A were rearranged by Maxwell’s rearrangement, losing C_3_O_2_, and a molecule of H_2_O was removed to form the fragment ion at *m/z* 451.0809. On the basis of the fragment ion at *m/z* 451.0809, if the bonds of 0/4 in ringⅠ-C were broken (-C_2_H_2_), the fragment ion at *m/z* 443.0390 was formed. And if the bonds of 1/2 were broken (-C_7_H_5_O_2_), the fragment ion at *m/z* 307.0585 was produced by losing an oxygen atom continuely. The fragment ion at *m/z* 417.0597 was formed as a result of the fractures of bond 0/2 in ringⅡ-C (-C_7_H_5_O_2_). Then H_2_O was lost, and the fragment ion at *m/z* 399.0490 was produced. On the basis of the fragment ion at *m/z* 417.0597, if C_3_O_2_ and H_2_O were lost, the fragment ion at *m/z* 331.0590 was formed. And if C_3_O_2_ and O were lost, the fragment ion at *m/z* 333.0378 can be obtained. The fragment ion of *m/z* 375.0496 was generated by the fracture of bonds 0/4 in ringⅡ-C (-C_9_H_6_O_3_), and dehydration was occurred to obtain the fragment ion at *m/z* 357.0376. AMF can be broken into monoflavone. After that, the two γH in ring A will undergo Maxwell’s rearrangement and lose C_3_O_2_, followed by the loss of C_2_H_2_O. After hydrogen rearrangement, the fragment ion at *m/z* 159.0433 would be obtained. And the fragment ion at *m/z* 117.0332 would be obtained by loss of C_2_H_2_O. The fragment ion at *m/z* 203.0328 were considered as the result of the fracture of bonds 0/3 in ringⅡ-C and bonds 1’/5’ in ringⅠ-B.

1. ***Metabolites of AMF***

In this study, a total of 34 metabolites of AMF were found. Metabolites were mainly distributed in feces, including 34 metabolites, and 3 metabolites were in urine (M4, M7, M17). No metabolite of AMF was found in bile or plasma.

*Oxidation.* Metabolites M1-M7 were a group of isomers with retention time of 5.80 min, 6.04 min, 6.63 min, 6.80 min, 7.11 min, 7.30 min and 7.65 min, respectively. The deprotonated molecular ions [M-H]^-^ were at *m/z* 553.0748, 553.0746, 553.0749, 553.0761, 553.0749, 553.0754 and 553.0754, respectively. Compared with the parent drug, they were all 16 Da larger than that of AMF, so it was speculated that they were products of oxidation reaction of AMF, and the molecular formula was C_30_H_18_O_11_.

Metabolite M1 was eluted at 5.80 min, and the deprotonated molecular ion [M-H]^-^ was at *m/z* 553.0748.The fragment ion at *m/z* 535.3241 was formed by the loss of H_2_O. The fragment ions at *m/z* 399.0500, 443.0401, 375.0502 and 333.0399 were consistent with those of AMF. The typical fragment ion at *m/z* 133.0282 was 16 Da larger than that of AMF at *m/z* 117.0332, so oxidation reaction was occurred in the position of C-5’ or C-6’of ringⅠ-B or C-5’ or C-6’ of ringⅡ-B. Because the polarity of M1 was the greatest in all the oxidation metabolites, we can infer that oxidation reaction occurred in the position of C-6’ of ringⅠ-B.

The retention time of metabolite M5 was 7.11 min, and the deprotonated molecular ions [M-H]^-^ was at *m/z* 553.0749. The fragment ion at *m/z* 535.3180 was formed by the loss of H_2_O. The fragment ions *m/z* 443.0385, 375.0501, 307.0207, 159.0451 and 117.0344 were all consistent with the fragment ions of AMF. The fragment ion at *m/z* 133.0284 was 16 Da larger than the ion of AMF at *m/z* 117.0344, so oxidation reaction was happened in the position of C-5’ or C-6’of ringⅠ-B or C-5’ or C-6’ of ringⅡ-B. Comparing the polarity and the retention time of the isomers in this group comprehensively, and the ClogP value of oxidation product at C-5’ of ringⅠ-B and C-5’、C-6’ of ring Ⅱ-B, it can be deduced that metabolite M5 may be the product of oxidation of AMF at C-5’ of ringⅡ-B.

Metabolite M6 was determined at 7.30 min, and the deprotonated molecular ions [M-H]^-^ was at *m/z* 553.0754. The secondary fragment ion at *m/z* 535.0650 was obtained by the loss of H_2_O. The fragment ions at *m/z* 417.0234, 159.0440 and 117.0388 were consistent with those of AMF. The typical ion at *m/z* 133.0316 was 16 Da larger than fragment ion at *m/z* 117.0388. The ClogP value of oxidation products at C-5’ of ringⅠ-B and C-6’ of ringⅡ-B was compared, and it can be deduced that M6 was likely to be the oxidation product at C-5’ of ringⅠ-B.

Metabolites M7 was extracted at 7.65 min, and the deprotonated molecular ion [M-H]^-^ was at *m/z* 553.0750. The secondary ions at *m/z* 417.0373, 443.0438, 375.0504, 159.0414 and 117.0130 were consistent with those of AMF. The fragment ions at *m/z* 467.0776 was produced by the loss of H_2_O and C_3_O_2_, therefore, oxidation was not going to happen in ringⅠ-A. Comparing the ClogP value of these oxidation metabolites, it can be concluded that M7 was the oxidation product of AMF at C-6 of ring Ⅱ-A.

The retention time of the metabolite M2 was 6.04 min, and the deprotonated molecular ion [M-H]^-^ was at *m/z* 553.0746. The secondary fragment ions at *m/z* 493.2843, 375.2439, 159.0452 and 117.0346 were consistent with the parent drug’s cracking fragments. Metabolites M3 and M4 was detected 6.63 min and 6.80 min, respectively. The deprotonated molecular ions [M-H]^-^ were at *m/z* 553.0749 and 553.0761. The fragment ion of M3 at *m/z* 485.0854 and the ion of M4 at *m/z* 485.0825 were formed by the loss of C_3_O_2_ in ringⅠ-A. Thus oxidation reaction did not occur in ringⅠ-A. Besides the identified metabolites of M1, M5, M6 and M7, comparing a few sites oxidation metabolites ClogP values, and according to the retention times, we can conclude that M2, M3, and M4 were the oxidation metabolites of AMF at C-6’ of ringⅡ-B, C-3 of ringⅠ-C and C-3 of ringⅡ-C , respectively.

*Methylation.* The retention times of metabolites M8~M13 were 9.39 min, 9.99 min, 10.51 min, 10.82 min, 13.25 min and 14.31 min, respectively. The deprotonated molecular ions [M-H]^-^ were at *m/z* 551.0967, 551.0975, 551.0952, 551.0978, 551.0969 and 551.0974, respectively, which were 14 Da larger than that of AMF. The molecular formula was C_31_H_20_O_10_. Methylation can occur on all the six hydroxyl groups of AMF, so six isomers would be produced.

The metabolite M8 was eluted at 9.39 min, and the deprotonated molecular ion [M-H]^-^ was at 551.0967. The secondary fragment ion at *m/z* 519.0699 was formed by the loss of two O in the parent ion, and ions at *m/z* 493.0529 and 117.0340 were consistent with the parent fragments. The characteristic fragment ions at *m/z* 431.0745, 413.0632 and 389.0636 were 14 Da larger than the cracked fragments of AMF at *m/z* 417.0597, 399.0490 and 375.0376, respectively. So methylation was taken place at 5-hydroxyl or 7-hydroxyl of ringⅠ-A or 5-hydroxyl of ringⅡ-A. Moreover, metabolites M8 had the greatest polarity in the six isomers, thus it was determined that metabolites M8 was formed by methylation at 5-hydroxyl of ringⅠ-A.

Metabolites M9, observed at 9.99 min, showed the deprotonated molecular ion [M-H]- at *m/z* 551.0975. The fragment ions at *m/z* 431.0744, 413.0631, and 389.0634 were 14 Da larger than the cracked fragments of AMF at *m/z* 417.0597, 399.0490 and 375.0376, respectively. So methylation was arisen at 7- hydroxyl of ringⅠ-A or 5- hydroxyl of ring Ⅱ-A. Because of its greater polarity, it can be concluded that methylation was arisen at 5- hydroxyl of ring Ⅱ-A.

Metabolites M10 and M11 were extracted at 10.51 min and 10.82 min, and displayed deprotonated molecular ion [M-H]^-^ at 551.0978 and 551.0951. Their secondary fragment ions at *m/z* 431.0749 and 457.0517 were 14 Da larger than the fragment ions of AMF at *m/z* 443.0390 and 417.0597, respectively. It indicated methylation was occurred at 7-hydroxyl of ringⅡ-A or 4’-hydroxyl of ringⅠ-B. By comparing the polarity of M10 and M11, and the ClogP value of the two methylation products, it could be deduced that M10 and M11 were the methylation products of AMF at 7-hydroxyl of ringⅡ-A ring and 4’-hydroxyl of ringⅠ-B, respectively.

The retention time of Metabolites M12 was 13.25 min, and the deprotonated molecular ion [M-H]^-^ was at *m/z* 551.0969. The typical fragment ion at *m/z* 483.1061 was the result of loss of C_3_O_2_ in ringⅠ-A. Therefore, methylation reaction could not occur in ringⅠ-A. Thus, M12 was the methylation product of AMF at 4’-hydroxyl of ringⅡ-B.

Metabolite M13 had a retention time of 14.31 min, and its deprotonated molecular ion [M-H]^-^ was at *m/z* 551.0974. The secondary fragment ions *m/z* 507.1041, 413.0503 and 389.0608 were 14 Da larger than the parent fragments at *m/z* 493.0915, 399.0490 and 375.0496, respectively. Fragment ions at *m/z* 375.0558 and 117.0343 were identical to the parent fragments. Because its polarity was the weakest among the six isomers, therefore, M13 was the metabolite of methylation of 7-hydroxyl in ring I-A.

*Oxidation and methylation.* Metabolites M14~M20 were all found at *m/z* 567, 30 Da larger than that of AMF. It was presumed that they were metabolites of oxidation and methylation reaction of AMF, and the molecular formula was C_31_H_20_O_11_.

The retention time of metabolite M15 was 7.21 min, and the deprotonated molecular ion [M-H]^-^ was at *m/z* 567.0908. The secondary fragment ion at *m/z* 499.3021 was 68 Da smaller than that of the parent ion, which indicated that oxidation and methylation did not occur on ring I-A, and the fragment ion at *m/z* 141.0680 was a fragment with four hydroxyl groups on the benzene ring, so it was speculated that oxidation and methylation occurred on ring II-A. Metabolite M15 was the metabolite of oxidation and methylation of parent drug at C-6 of ring II-A.

Metabolite M14 was detected at 6.53 min, and showed the quasi-molecular ion at [M-H]^-^ at *m/z* 567.0904. The retention time was earlier than that of metabolite M15. It indicated that oxidation and methylation was taken place at C-2’ of ring I-B or C-6’ of ring I-A. Considering the effect of steric hindrance, it was speculated that M14 may be the product of oxidation and methylation at C-6’ of ring I-A.

Metabolites M16-M20 were retained for 7.36 min, 8.08 min, 8.37 min, 8.64 min and 10.99 min, respectively. The deprotonated molecular ion [M-H]^-^ were at *m/z* 567.0905, 567.0917, 567.0909, 567.0909 and 567.0929, respectively. Metabolites M16, M17 and M19 all had secondary fragment ion at *m/z* 499, indicating that oxidation and methylation could not occur on ring I-A. The fragment ion of M16 at *m/z* 189.0547 was 30 Da larger than that of AMF at *m/z* 159.0433, indicating that oxidation and methylation occurred on I-B or ring II-B. The fragment ion of metabolite M17 at *m/z* 147.0438 was 30 Da larger than that of AMF at *m/z* 117.0332, indicating that oxidation and methylation also occurred on I-B or ring II-B. The secondary fragment ions at *m/z* 473.0607 and 447.0701 of metabolite M20 were 30 Da larger than those of AMF at *m/z* 443.0390 and 417.0597, respectively, indicating that oxidation and methylation occurred on ring I-B or ring II-A. The ion at *m/z* 146.9619 was 30 Da larger than the ion at 117.0332 of AMF, so oxidation and methylation occurred at C-5’ or C-6’ of ring I-B. Comparing the ClogP values of oxidation and methylation metabolites at C-6’, C-8’ of ring Ⅰ-A, C-2’, C-5’, C-6’ of ring Ⅰ-B, C-5’ and C-6’ of ring Ⅱ-B, it could be inferred that M16~M20 was the metabolite of oxidation and methylation at C-6’ of ring II-B, C-6’ of ringⅠ-B, C-8’ of ringⅠ-A, C-5’ of ring II-B and C-5’ of ringⅠ-B, respectively.

*Hydrogenation*. Metabolites M21 and M 22, with retention time of 8.47 min and 8.81 min, deprotonated molecular ions [M-H]^-^ at *m/z* 539.0958 and 539.0960, respectively, were 2 Da larger than that of AMF. Therefore, it was presumed that AMF had undergone hydrogenation reaction, and the molecular formula was C_30_H_20_O_10_. The secondary fragment ion at *m/z* 135.0439 of metabolite M21 indicated that the hydrogenation reaction occurred at C=C of ring I-C or ring II-C. The secondary fragment ion at *m/z* 119.0493 of metabolite M22 was 2 Da larger than the fragment ion at *m/z* 117.0332 of AMF, which also indicated that hydrogenation occurred at C=C of ring I-C or ring II-C. Therefore, by comparing the retention time of M21 and M22 and the ClogP values of the two hydrogenation products, it was inferred that the metabolites M21 and M22 were products of hydrogenation reaction of C=C of ring II-C and ring I-C, respectively.

*Di-hydrogenation*. Metabolite M23 was appeared at 8.62 min, and the deprotonated molecular ion [M-H]^-^ was at *m/z* 541.1117, which was 4 Da larger than that of AMF. It was speculated that AMF had a di-hydrogenation reaction, and the molecular formula was C_20_H_22_O_10_. The typical ion, *m/z* 161.0603, was 2 Da larger than the fragment ion at *m/z* 159.0433 of AMF. The result showed that there was a hydrogenation reaction in ring I-C and II-C of AMF. The characteristic ions at *m/z* 497.1198 and 421.0607 indicated that the hydrogenation reaction of ring II-C occurred on the hydroxyl group; the fragment ion at *m/z* 353.0641 indicated that the hydrogenation reaction of ring I-C occurred on C=C. So the metabolite M23 was the hydrogenation product of the hydroxyl of ring II-C and C=C of ring I-C of AMF.

The retention time of metabolite M24 was 10.49 min, and the deprotonated molecular ion [M-H]^-^ was at *m/z* 521.0862, which was 16 Da smaller than that of AMF. Therefore, it was presumed that the mother drug had lost an oxygen atom and the molecular formula was C_30_H_18_O_9_. The secondary fragment ion *m/z* 503.3300 was formed by the loss of a molecule of H_2_O from the parent ion. And the typical fragment ions at *m/z* 399.0482 and 375.0492 illustrated that the lost oxygen atom was located in ring II-B. Therefore, it was inferred that the metabolite M24 was formed by the loss of O from the ring II-B of AMF.

*Acetylation*. Metabolites M25~M30 were a group of isomers. Excimer ion peaks [M-H]^-^ were at *m/z* 579.0909, 579.0915, 579.0902, 579.0909, 579.0922 and 579.0922, which were 42 Da larger than that of AMF. It was presumed that AMF had acetylation reaction, and the molecular formula was C_32_H_20_O_11_.

Metabolite M25 was extracted at 9.27 min, and the excimer ion [M-H]^-^ was at *m/z* 579.0909. The secondary fragment ions at *m/z* 485.0482 and 493.0903 were 42 Da larger than the fragment ions at *m/z* 451.0809 and 443.0390 of AMF. It showed that the acetylation reaction occurred on the 5-hydroxyl or 4’- hydroxyl of ring II-A. Because of the greatest polarity in the six isomers, the metabolite M25 was deduced to be the product of acetylation of 4’-hydroxyl of ring II-B.

Metabolites M26-M28 were eluted at 9.95 min, 10.39 min and 10.63 min, respectively. The corresponding deprotonated molecular ions [M-H]^-^ were at *m/z* 579.0915, 579.0902 and 579.090909. They all had the secondary fragment ion at *m/z* 561. Therefore, the acetylation reaction could not occur on the 4’-hydroxyl of ring I-B and 7'-hydroxyl of ring II-A. Comparing the retention times of three metabolites and acetylated metabolites of 5-hydroxyl, 7-hydroxyl of ring I-A and 5- hydroxyl of ring Ⅱ-A, it could be inferred that the metabolites M26~M28 were the products of acetylation of AMF at 7-hydroxyl of ring I-A, 5- hydroxyl of ring II-A and 5- hydroxyl of ring I-A, respectively. The retention times of metabolites M29 and M30 were 12.31 min and 13.04 min, respectively, and the excimer ion peaks [M-H]^-^ were both at *m/z* 579.0922. Based on the retention time and ClogP values of acetylation products at 4’- hydroxyl of ring I-B and 7’-hydroxyl of ring II-A, it was inferred that M29 and M30 were metabolites of acetylation of 7’-hydroxyl of ring II-A and 4’- hydroxyl of ring I-B, respectively.

*Loss of O and Glycine conjugation*. Metabolite M31 was detected at 7.53 min, and the excimer ion peak [M-H]^-^ was at *m/z* 578.1060, which was 41 Da larger than AMF. It was presumed that the parent drug lost an oxygen atom and combined with a glycine. The molecular formula was C_32_H_21_NO_10_. The secondary fragment ions at *m/z* 417.0617, 399.0487 and 375.0503 were identical with those of AMF, indicating that the lost oxygen atom was located at 4’-hydroxyl of II-A ring. Moreover, the typical fragment ions *m/z* 306.1181 and 150.0375 indicated that the glycine was bound to 5-hydroxyl of ring II-A. Therefore, it was inferred that M31 was a metabolite formed by the combination of 5-hydroxyl of ring II-A with glycine after the loss of O at 4’- hydroxyl of ring II-B.

*Loss of O and N-Acetylation*. The retention time of the metabolite M32 was 10.88 min, the excimer ion [M-H]^-^ was at *m/z* 563.0960, which was 26 Da larger than AMF. It was speculated that AMF lost an oxygen atom and acetylated, and the molecular formula was C_32_H_20_O_10_. The secondary fragment ion at *m/z* 545.0876 was formed by the loss of a molecule of H_2_O; and the fragment ion at *m/z* 357.0747 indicated that the lost O was at 4'-hydroxyl of ring II-B. The typical fragment ion at *m/z* 383.0528 was caused by RDA reaction of part I and the loss of CO of ring II-C. Therefore, the acetylation was taken place on 5-hydroxyl of ring II-A. Thus, it was inferred that M32 was a metabolite obtained by losing the O of 4’-hydroxyl of ring II-B and acetylating 5’-hydroxyl of ring II-A.

*Loss of O and hydrogenation*. The metabolite M33 had a retention time of 10.72 min, excimer ion peak [M-H] ^–^ at *m/z* 523.1013, which was 14 Da smaller than AMF. It was presumed that AMF was deoxidized and hydrogenated, and the molecular formula was C_30_H_20_O_9_. The fragment ions at *m/z* 417.0596, 375.0485 and 307.0589 were identical with that of AMF. It showed that the lost oxygen atom was located on 4’-hydroxyl of ring II-B and the hydrogenation reaction occurred on C=C of ring II-C. Therefore, it was inferred that M33 was a metabolite of AMF which lost the O of 4’-hydroxyl of ring II-B and hydrogenated at C=C of ring II-C.

*Internal Hydrolysis*. Metabolite M34 was found at 8.44 min, and detected at *m/z* 555.0918, which was 18 Da larger than AMF. Therefore, it was presumed that internal hydrolysis was occurred on AMF, and the elemental compositions was C_30_H_20_O_11_. The fragment ion at *m/z* 509.2884 was 46 Da smaller than the parent ion, which was formed by the loss of CO and H_2_O. And the typical ion at *m/z* 487.3033 was 68 Da smaller than the parent ion, indicating that a molecular of C_3_O_2_ was lost on the basis of the parent ion. While the characteristic fragment ion at *m/z* 403.0834 was formed by the RDA reaction of the part Ⅰ, so it could be inferred that M34 was formed by the hydrolysis at C=C of ring II-C.

1. ***Metabolite analysis of AMF-loaded TPGS/soluplus mixed micelles***

Fourteen metabolites of AMF-loaded micelles were found in rats, including three in plasma (N7, N8, N13), six in urine (N2, N3, N5, N7, N10, N11), and 11 in feces (N1, N4-N12, N14).

*Oxidation*. The metabolites N1, N2 and N3 were a group of isomers with retention time of 6.04 min, 7.30 min and 7.65 min, excimer ion [M-H]^-^ at *m/z* 553.0774, 553.0774 and 553.0777, respectively, which were 16 Da larger than that of AMF. It was presumed that oxidation reaction was undergone, and the molecular formula was C_30_H_18_O_11_. The fragment ions at *m/z* 417.0608, 375.0505 and 331.0602 of N1 were consistent with the fragments of N0; the fragment ion at *m/z* 485.0855 was produced by the loss of C_3_O_2_, so the oxidation reaction could not occur on ring I-A. The the fragment ion at *m/z* 133.0285 indicated that the oxidation reaction occurred at C-5’, C-6’ of ring I-B or C-5’, C-6’ of Ⅱ-B. By comparing the retention time and the structure predicted by Metabolite Pilot^TM^ 2.0 software, it was inferred that N1 was the oxidation product of N0 at C-6’ of ring II-B, which was the same as M2. Both metabolites N2 and N3 had the secondary fragment ion at *m/z* 485, indicating that the oxidation reaction did not occur on ring I-A. The fragment ions at *m/z* 433.0500 and 174.9532 of N2 indicated that the oxidation reaction occurred at the C-5’or C-6’ of ring I-B, and the fragment ion at *m/z* 347.0530 of N3 indicated that the oxidation reaction happened on ring I-B or ring II-A. By comparing the retention time of metabolites with those of AMF, based on the structure predicted by Metabolite Pilot^TM^ 2.0 software, it was inferred that N2 and N3 were metabolites of oxidation reaction at C-5’ of ring I-B and C-6 of ring II-A, respectively. N2 and N3 were the same as M6 and M7, respectively.

*Methylation*. Metabolite N4~N8 was a group of isomers with retention time of 9.39 min, 9.99 min, 10.51 min, 10.82 min and 13.25 min. They all had the excimer ion [M-H]^-^ at *m/z* 551, which was 14 Da higher than that of N0. It was speculated that methylation reaction was taken place, and the molecular formula was C_31_H_20_O_10_.

Metabolite N4 was detected at 9.39 min, and the excimer ion peak [M-H]^-^ was at *m/z* 551.0980. The fragment ion at *m/z* 519.0735 was formed by the loss of two oxygen atoms. The fragments at *m/z* 431.0764 and 389.0635 were 14 Da larger than that at *m/z* 417.0597 and 375.0376 of N0, respectively. By comparing the retention time of N4 with the metabolites of M0, it was determined that the metabolite N4 was obtained by methylation of N0 at 5-hydroxyl of ring I-A, which was the same as the metabolite M8 of AMF.

Metabolite N5 was eluted at 9.99 min, with the excimer ion [M-H]^-^ at *m/z* 551.0986. The secondary fragment ions at *m/z* 519.0757, *m/z* 375.0522 and *m/z* 159.0445 were identical with the fragments of N0. The characteristic fragment at *m/z* 389.0677 was 14 Da higher than that of N0 at *m/z* 375.0376, indicating that methylation did not occur on ring II-B. The fragment ions at *m/z* 533.3134 and 483.085 were respectively formed by the loss of H_2_O and C_3_O_2_. So it was inferred that the metabolite M9 was the product of methylation of N0 on 5-hydroxyl of ring II-A, which was the same as the metabolite M9.

Metabolite N6 was determined at 10.51 min, showed the excimer ion peak at *m/z* 551.0976. The fragment ions at *m/z* 389.0667 was 14 Da larger than that of N0 at *m/z* 375.0376, indicating that methylation did not occur on ring II-B. The fragment at *m/z* 483.1010 was 68 Da smaller than that of N0, formed by the loss of C_3_O_2_, indicating that methylation reaction could not occur on ring I-A. And the fragment ion at *m/z* 190.9966 illustrated that the methylation occurred on ring II-A. Therefore, it was deduced that N6 was the product of methylation on 7-hydroxyl of ring II-A of N0, which was the same as M10.

Metabolite N7 was appeared at 10.82 min, and the deprotonated molecular ion [M-H]^-^ was at *m/z* 551.0980. The fragment ion at *m/z* 389.0686 was 14 Da higher than that at *m/z* 375.0376 of N0, indicating that methylation did not occur on ring II-B. The fragment at *m/z* 483.0173 was 68 Da smaller than N0, formed by the loss of C_3_O_2_, so the methylation reaction couldn’t occur on ring I-A. The fragment at *m/z* 345.0753 was 14 Da larger than tha of N0, so it was inferred that N7 was the metabolite of methylation of N0 at the 4’- hydroxyl of ring I-B, which was the same as M11.

Metabolite N8 was retained for 13.25 min in the chromatography, and the excimer ion peak was appeared at *m/z* 551.0985. The secondary fragment ion at m/z 483.1087 demonstrated that methylation did not occur on ring I-A. Therefore, it was speculated that N8 was the metabolite of methylation at 4’- hydroxyl of ring II-B of N0, which was the same as the metabolite M12.

*Oxidation and methylation*. The metabolites N9 and N10 were a pair of isomers with excimer ion peaks [M-H]^-^ at *m/z* 567.0934 and *m/z* 567.0935, respectively. Both of them had the fragment ion at *m/z* 189, which was 30 Da larger than the fragments of N0 at 159.0433. Therefore, it was presumed that oxidation and methylation was occurred on ring I-B or ring II-B. By comparing with the retention time of the metabolites of AMF, it could be determined that N9 and N10 were C-6’ of ring II-B and C-6’ of ringⅠ-B, and were the same as those of M16 and M17, respectively.

*Loss of O*. Metabolite N11 had the retention time of 10.49 min, and the excimer ion peak at *m/z* 521.0882, which was 16 Da smaller than that of N0. Therefore, it was presumed that the parent drug lost an oxygen atom and its molecular formula was C_30_H_18_O_9_. The fragment ion at *m/z* 503.3361 was formed by the removal of H_2_O, so the 4’-hydroxyl of ring I-B and 7’-hydroxyl of ring II-A could not be removed. Both *m/z* 399.0497 and *m/z* 375.0523 indicated that the lost oxygen atom was located on ring II-B. Therefore, it was inferred that N11 was a metabolite formed by removing an oxygen atom from the hydroxyl group of ring II-B, which was the same as M24.

*Hydrogenation*. The retention time of the metabolite N12 was 8.81 min, the excimer ion peak [M-H]^-^ was at *m/z* 539.0985, which was 2 Da larger than that of N0. It was speculated that the mother drug underwent hydrogenation reaction, and the molecular formula was C_30_H_20_O_10_. The secondary fragment ion at *m/z* 119.0484 was 2 Da larger than the cracked fragment at *m/z* 117.0332 of N0, so it was presumed that the hydrogenation reaction was occurred on C=C of ring I-C or ring II-C, and the fragments at *m/z* 419.0536 and 309.0391 were both 2 Da higher than that of N0 at *m/z* 417.0597 and 307.0585, respectively. Therefore, it was inferred that the hydrogenation reaction was occurred on the C=C of ring I-C, which was the same as the metabolite M22.

*Phosphorylation*. Metabolite N13 was eluted at 1.67 min, and the excimer ion [M-H]^-^ was at *m/z* 617.0467, which was 80 Da larger than that of the parent drug. It was speculated that the mother drug had been phosphorylated, and the molecular formula was C_30_H_19_O_13_P. The characteristic fragment ion at *m/z* 446.9946 was formed by RDA cleavage of N0 on ring I-C and loss of H_2_O from 4’-hydroxyl of ring I-B and 7’-hydroxyl of ring II-A. Therefore, the site of phosphorylation may be the 5’-hydroxyl of ring II-A or 4’-hydroxyl of ring II-B. Considering the influence of steric hindrance and the hydrogen bonding between the 5’-hydroxyl of ring II-A and the carbonyl group of ring II-C, N13 was speculated to be a metabolite of N0 which was phosphorylated on the 4’-hydroxyl of ring II-B.

*Loss of O and Glycine conjugation*. Metabolite N14 was found at 7.53 min, and the excimer ion peak was appeared at *m/z* 578.1092, which was 41 Da larger than that of N0. It was forecasted that N0 was removed an oxygen atom and bound to glycine, and the molecular formula was C_32_H_21_NO_10_. The fragment ions at *m/z* 417.0627, *m/z* 399.0467, 375.0509 and 331.0601 were identical with the fragments of N0, indicating that the lost oxygen atoms were located at the 4’- hydroxyl of ring II-B. By comparing with the retention time of the metabolites of M0, it was speculated that N14 might be the metabolite of the 5-hydroxyl group of ring II-A combined with glycine after the removal of the 4’-hydroxyl of ring II-B from the parent drug. The product was the same as the metabolite M31.


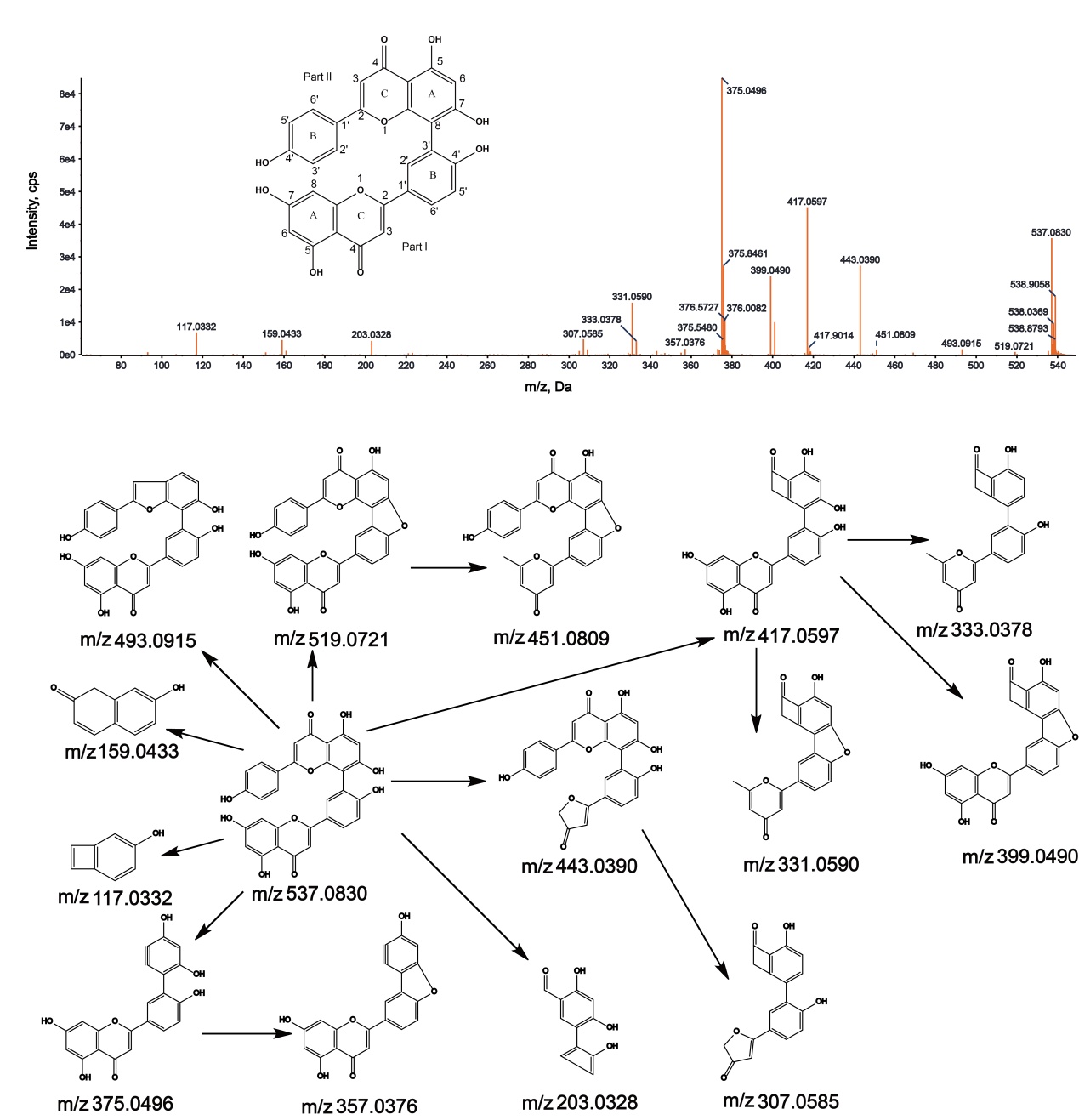


**Fig.1.** MS/MS spectrum of AMF and its predominant fragmentation pathways
